# Creutzfeldt-Jakob Disease Incidence, South Korea, 2001–2019

**DOI:** 10.3201/eid2809.212050

**Published:** 2022-09

**Authors:** Yong-Chan Kim, Byung-Hoon Jeong

**Affiliations:** Jeonbuk National University, Jeonju, South Korea

**Keywords:** Prions and related diseases, zoonoses, Creutzfeldt-Jakob disease, South Korea

## Abstract

We found increasing trends of Creutzfeldt-Jakob disease (CJD) cases and annual incidence in South Korea during 2001–2019. We noted relatively low (5.7%) distribution of familial CJD. An unusually high percentage (≈1%) of patients were in the 30–39 age group, which should prompt a preemptive CJD control system.

Prion diseases are fatal, irreversible, and transmissible brain proteinopathies caused by abnormal aggregated prion protein (PrP^Sc^) converted from normal prion protein (PrP^C^), which is encoded by the prion protein gene (*PRNP*) (*1*–*3*). The major type of human prion disease is Creutzfeldt-Jakob disease (CJD), and several countries have reported increasing trends in CJD cases and incidence (*4*). 

CJD is subdivided into 3 types: sporadic, familial, and iatrogenic (*5*–*7*). The most common type is sporadic CJD, which accounts for >85% of all CJD cases. Familial CJD accounts for 10%–15% of all cases and is caused by germline mutations of the human *PRNP* gene, including D178N-M129V, V180I, E200K, V203I, and M232R. Iatrogenic CJD accounts for <1% of all CJD cases and includes variant CJD, which is caused by ingestion of beef products from bovine spongiform encephalopathy–affected cattle (*7*–*9*).

In South Korea, the Korea Centers for Disease Control and Prevention (KCDC) has operated the CJD surveillance system and issued epidemiologic reports since 2001. In addition, except for variant CJD, the surveillance system has reported all 3 types of CJD. Furthermore, CJD has been reported annually and has exhibited increasing trends in case numbers. However, no long-term estimation of the various characteristics of CJD patients, including onset age, sex, germline mutations of familial CJD, or overall CJD incidence are available for South Korea. We conducted a study on CJD cases in South Korea from 2001–2019 to assess case count and incidence trends.

## The Study

We collected data on CJD patients from Statistics Korea (KOSTAT) (https://kostat.go.kr) and epidemiologic reports issued by the KCDC (https://www.kdca.go.kr). We analyzed the characteristics of CJD patients in South Korea. In addition, we obtained the global incidence of sporadic CJD from previous studies and compared these with the incidence in South Korea (*4*). 

CJD diagnosis was performed according to guidelines from the World Health Organization in South Korea (*10*). During 2001–November 2010, CJD was controlled through a sample monitoring system centered in medical institutions in the country and KCDC collected the CJD-related data. Beginning December 2010, CJD was designated as a statutory infectious disease, and KCDC conducts a complete epidemiologic investigation and monitors suspected patients. CJD death rates in South Korea have not been available. 

The number of possible, probable, and definite CJD diagnoses was not available during 2001–2015. Among all cases during 2016–2019, KCDC reported 6 (3.2%) possible CJD cases, 179 (95.2%) probable cases, and 3 (1.6%) definite cases. Genetic testing was performed on all patients with diagnosed CJD. 

We obtained population data stratified by age, region, sex, and year from KOSTAT. We used χ^2^ test to determine statistically significant differences and performed calculations in SAS version 9.4 (SAS Institute Inc., https://www.sas.com).

**Figure 1:**
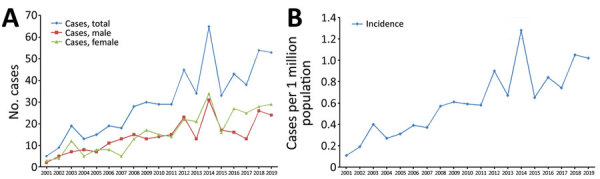
Annual incidence of Creutzfeldt-Jakob disease, South Korea, 2001–2019. A) Total number of reported cases per year and sex. B) Annual incidence per million population.

During 2001–2019, a total of 579 CJD cases were reported in South Korea (Table). Of note, the annual number of cases and CJD incidence in South Korea exhibited an upward trend (Figure 1, Table). Among 579 cases, 545 (94.13%) were sporadic, 33 (5.7%) were familial, and 1 (0.17%) was iatrogenic. Variant CJD was not reported in Korea during 2001–2019 (Table). During 2008–2019, a total of 31 familial CJD cases were reported, as were 5 types of germline mutations of the *PRNP* gene, including D178N-M129V, V180I, E200K, V203I and M232R (Appendix Table 1, Figure 1).

**Table:** Annual cases and incidence of Creutzfeldt-Jakob disease, Korea, 2001–2019

Year	Patients, no. (%)	Sex, no. (%)		p value	Subtypes, no. (%)				Total population	Incidence‡
		M	F		Sporadic	Iatrogenic†	Variant	Familial		
2001	5 (0.86)	2 (40)	3 (60)	0.7498	5 (100)	0	0	0	47,370,164	0.11
2002	9 (1.55)	5 (55.56)	4 (44.44)	0.6162	9 (100)	0	0	0	47,644,736	0.19
2003	19 (3.28)	7 (36.84)	12 (63.16)	0.3756	19 (100)	0	0	0	47,892,330	0.40
2004	13 (2.25)	8 (61.54)	5 (38.46)	0.3042	12 (92.31)	0	0	1 (7.69)	48,082,519	0.27
2005	15 (2.59)	7 (46.67)	8 (53.33)	0.9705	14 (93.33)	0	0	1 (6.67)	48,184,561	0.31
2006	19 (3.28)	11 (57.89)	8 (42.11)	0.3561	19 (100)	0	0	0	48,438,292	0.39
2007	18 (3.11)	13 (72.22)	5 (27.78)	**0.0360**	18 (100)	0	0	0	48,683,638	0.37
2008	28 (4.84)	15 (53.57)	13 (46.43)	0.5063	25 (89.29)	0	0	3 (10.71)	49,054,708	0.57
2009	30 (5.18)	13 (43.33)	17 (56.67)	0.6829	29 (96.67)	0	0	1 (3.33)	49,307,835	0.61
2010	29 (5.01)	14 (48.28)	15 (51.72)	0.9057	28 (96.55)	1 (3.45)	0	0	49,554,112	0.59
2011	29 (5.01)	15 (51.72)	14 (48.28)	0.6302	26 (89.66)	0	0	3 (10.34)	49,936,638	0.58
2012	45 (7.77)	23 (51.11)	22 (48.89)	0.6083	42 (93.33)	0	0	3 (6.67)	50,199,853	0.90
2013	34 (5.87)	13 (38.24)	21 (61.76)	0.3112	31 (91.18)	0	0	3 (8.82)	50,428,893	0.67
2014	65 (11.23)	31 (47.69)	34 (52.31)	0.9338	62 (95.38)	0	0	3 (4.62)	50,746,659	1.28
2015	33 (5.70)	17 (51.52)	16 (48.48)	0.6252	33 (100)	0	0	0	51,014,947	0.65
2016	43 (7.43)	16 (37.21)	27 (62.79)	0.2073	173 (92.02)§	0	0	15 (7.98)	51,217,803	0.84
2017	38 (6.56)	13 (34.21)	25 (65.79)	0.1213	–	–	–	–	51,361,911	0.74
2018	54 (9.33)	26 (48.15)	28 (51.85)	0.8883	–	–	–	–	51,606,633	1.05
2019	53 (9.15)	24 (45.28)	29 (54.72)	0.7943	–	–	–	–	51,709,098	1.02
Total	579 (100)	273 (47.15)	306 (52.85)	–	545 (94.13)	1 (0.17)	0	33 (5.70)	–	–

*CJD includes possible, probable, and definite CJD diagnoses. Bold text indicates statistical significance (p<0.05). CJD, Creutzfeldt-Jakob disease; –, not applicable. †The source of transmission for the iatrogenic CJD case is dura mater graft. ‡Cases per million population §Reported cases and subtypes of CJD in South Korea, 2016–2019.

**Figure 2:**
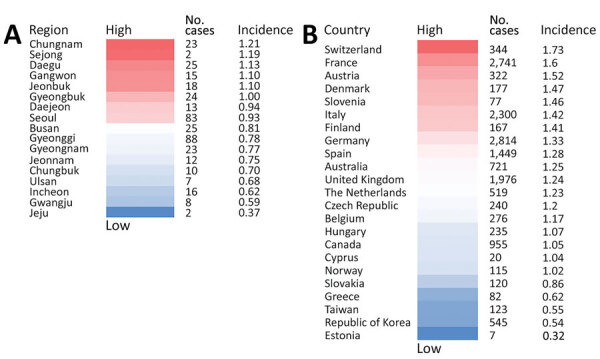
Creutzfeldt-Jakob disease (CJD) incidence, South Korea and globally, 2001–2019. A) Number of cases and incidence per million persons in cities and provinces of South Korea, including probable, possible, and definite CJD diagnoses. B) Global number of sporadic CJD cases and incidence per million population by country (*4*). Sporadic CJD incidence for South Korea is from this study.

During 2011–2019, a total of 394 cases of CJD were reported in South Korea; 162 (41.12%) among persons >70 years of age, 129 (32.74%) among persons 60–69 years of age, 75 (19.04%) among persons 50–59 years of age, 23 (5.84%) among persons 40–49 years of age, and 5 (1.27%) among persons 30–39 years of age (AppendixTable 2, Figure 2). Of note, the distribution of CJD cases by age in South Korea was significantly different in 2011 compared with 2011–2019 (p<0.05). In particular, the distribution of CJD in the 40–49 year age group in 2011 (17.24%) was ≈2.9 times higher than that of the 40–49 age group during 2011–2019 (5.84%).

Overall, Gyeonggi Province had the highest number of CJD cases (88, 22.34%), along with the cities of Seoul (83 cases, 21.07%), Busan (25 cases, 6.35%), and Daegu (25 cases, 6.35%). Sejong (2 cases, 0.51%) and Jeju (2 cases, 0.51%) had the fewest CJD cases, and Ulsan (7 cases, 1.78%) and Gwanju (8 cases, 2.03%) also had fewer cases than other cities (Appendix Table 3). Chungnam Province had the highest CJD incidence (1.21 cases per million population), along with the cities of Sejong (1.19 cases per million population) and Daegu (1.13 cases per million population). The city of Jeju had the lowest CJD incidence (0.37 cases per million population), and Gwangju (0.59 cases per million population) and Incheon (0.62 cases per million population) also had low incidence (Figure 2, panel A).

We compared the incidence of sporadic CJD in South Korea with the global incidence. Switzerland had the highest incidence (1.73 cases per million population), but incidence was also high in France (1.6 cases per million population) and Austria (1.52 cases per million population). Estonia had the lowest incidence of sporadic CJD at 0.32 cases per million population. By comparison, incidence of sporadic CJD in South Korea was extremely low, 0.54 cases per million population (Figure 2, panel B).

## Conclusions

In this study, we found increasing trends in CJD rates in South Korea during 2001–2019. These increasing trends might be the result of advanced diagnostic technology and capacity, more neurologic specialists, an increase in the average age of the population, or a combination of these factors. In addition, designation of CJD as a statutory infectious disease might have affected increased reporting. Nonetheless, further research could investigate the CJD incidence trend through the country’s high-level surveillance system. 

In addition, we observed that ≈1% of CJD patients in South Korea were in the 30–39-year age group (Appendix Figure 2). Because the average age of onset of familial CJD is over 50, younger CJD patients in the 30–39-year age group are extremely unusual (*11*,*12*). However, the exact subtypes of CJD (sporadic or variant) for each CJD patient in this age group were not available. Previous studies have reported variant CJD occurring at a relatively younger age than familial and sporadic CJD (7–9). Thus, further in-depth investigation of the subtypes of CJD among younger age groups is essential to determine causes of CJD in this age group and devise prevention strategies.

In conclusion, we identified increasing trends of CJD cases and incidence in South Korea during 2001–2019. We investigated CJD cases by subtype and found a relatively low (5.7%) distribution of familial CJD. Of note, sporadic CJD incidence was only 0.54 cases per million persons. In addition, we investigated the distribution of CJD patients by sex, age group, and region and found the highest incidence in the Chungnam region. A comprehensive long-term investigation could shed light on the characteristics of CJD in South Korea and help KCDC construct a preemptive control system.

Appendix Additional information on incidence of Creutzfeldt-Jakob disease, South Korea, 2001–2019.
