# Supplementary material for: Creutzfeldt-Jakob Disease Incidence, South Korea, 2001–2019
Source: Emerg Infect Dis. 2022 Sep;28(9):1863–6. doi: 10.3201/eid2809.212050 (PMC9423913; doi:10.3201/eid2809.212050)
Supplement: Appendix — Additional information on incidence of Creutzfeldt-Jakob disease, South Korea, 2001–2019. [file 21-2050-Techapp-s1.pdf]

Article DOI: https://doi.org/10.3201/eid2809.212050

Creutzfeldt-Jakob Disease Incidence, South Korea, 2001–2019

Appendix

Appendix Table 1. Classification of germline mutations of familial Creutzfeldt-Jakob disease, South Korea, 2008–2019

		Mutation, no. (%)				
Year	Total, no. (%)	D178N-M129V	V180I	E200K	V203I	M232R
2008–2010	4 (12.90)	0	1 (25)	1 (25)	1 (25)	1 (25)
2011–2015	12 (38.71)	1 (8.33)	5 (41.67)	6 (50)	0	0
2016–2019	15 (48.39)	0	5 (33.33)	4 (26.67)	0	6 (40)
Total	31 (100)	1 (3.23)	11 (35.48)	11 (35.48)	1 (3.23)	7 (22.58)

Appendix Table 2. Annual number of Creutzfeldt-Jakob disease cases by age group, South Korea, 2011–2019

		Age group, y; no. (%)					
Year	Total, no. (%)	30–39	40–49	50–59	60–69	≥70	p value
2011	29 (7.36)	0	5 (17.24)	3 (10.34)	14 (48.28)	7 (24.14)	**0.0307**
2012	45 (11.42)	2 (4.44)	2 (4.44)	9 (20)	13 (28.89)	19 (42.22)	0.5733
2013	34 (8.63)	1 (2.94)	2 (5.88)	6 (17.65)	10 (29.41)	15 (44.12)	0.9342
2014	65 (16.5)	0	2 (3.08)	16 (24.62)	18 (27.69)	29 (44.62)	0.5429
2015	33 (8.38)	0	0	5 (15.15)	14 (42.42)	14 (42.42)	0.4847
2016	43 (10.91)	0	4 (9.3)	8 (18.6)	10 (23.26)	21 (48.84)	0.5660
2017	38 (9.64)	0	2 (5.26)	9 (23.68)	11 (28.95)	16 (42.11)	0.9022
2018	54 (13.71)	1 (1.85)	3 (5.56)	10 (16.67)	20 (35.19)	20 (38.89)	0.9640
2019	53 (13.45)	1 (1.89)	3 (5.66)	9 (16.98)	19 (35.85)	21 (39.62)	0.9823
Total	394 (100)	5 (1.27)	23 (5.84)	75 (19.04)	129 (32.74)	162 (41.12)	–

*Bold text indicates statistical significance ($p<0.05$). –, not applicable.

Appendix Table 3. Annual number of Creutzfeldt-Jakob disease cases by city and province, South Korea, 2011–2019

Year	Total, no. (%)	Seoul	Busan	Daegu	Incheon	Gwanju	Daejeon	Ulsan	Sejong	Gyeonggi	Gangwon	Chungbuk	Chungnam	Jeonbuk	Jeonnam	Gyeongbuk	Gyeongnam	Jeju
2011	29 (7.36)	7 (24.14)	0	4 (13.79)	3 (10.34)	1 (3.45)	1 (3.45)	0	0	7 (24.14)	1 (3.45)	0	2 (6.9)	1 (3.45)	1 (3.45)	0	0	1 (3.45)
2012	45 (11.42)	9 (20)	2 (4.44)	3 (6.67)	1 (2.22)	2 (4.44)	2 (4.44)	1 (2.22)	0	10 (22.22)	3 (6.67)	1 (2.22)	4 (8.89)	3 (6.67)	2 (4.44)	0	2 (4.44)	0
2013	34 (8.63)	7 (20.59)	4 (11.76)	5 (14.71)	1 (2.94)	1 (2.94)	1 (2.94)	1 (2.94)	0	1 (2.94)	1 (2.94)	1 (2.94)	4 (11.76)	1 (2.94)	1 (2.94)	3 (8.82)	2 (5.88)	0
2014	65 (16.5)	12 (18.46)	2 (3.08)	3 (4.62)	3 (4.62)	0	2 (3.08)	1 (1.54)	0	15 (23.08)	2 (3.08)	3 (4.62)	6 (9.23)	1 (1.54)	1 (1.54)	9 (13.85)	5 (7.69)	0
2015	33 (8.38)	5 (15.15)	4 (12.12)	2 (6.06)	1 (3.03)	0	1 (3.03)	0	0	8 (24.24)	3 (9.09)	0	1 (3.03)	2 (6.06)	2 (6.06)	1 (3.03)	2 (6.06)	1 (3.03)
2016	43 (10.91)	13 (30.23)	3 (6.98)	3 (6.98)	3 (6.98)	1 (2.33)	1 (2.33)	1 (2.33)	1 (2.33)	8 (18.6)	0	1 (2.33)	3 (6.98)	2 (4.65)	0	1 (2.33)	2 (4.65)	0
2017	38 (9.64)	10 (26.32)	3 (7.89)	2 (5.26)	1 (2.63)	2 (5.26)	1 (2.63)	0	1 (2.63)	8 (21.05)	1 (2.63)	2 (5.26)	1 (2.63)	0	1 (2.63)	2 (5.26)	3 (7.89)	0
2018	54 (13.71)	12 (22.22)	5 (9.26)	2 (3.7)	1 (1.85)	0	1 (1.85)	3 (5.56)	0	14 (25.93)	2 (3.7)	0	2 (3.7)	3 (5.56)	0	4 (7.41)	5 (9.26)	0
2019	53 (13.45)	8 (15.09)	2 (3.77)	1 (1.89)	2 (3.77)	1 (1.89)	3 (5.66)	0	0	17 (32.08)	2 (3.77)	2 (3.77)	0	5 (9.43)	4 (7.55)	4 (7.55)	2 (3.77)	0
Total†	394 (100)	83 (21.07)	25 (6.35)	25 (6.35)	16 (4.06)	8 (2.03)	13 (3.3)	7 (1.78)	2 (0.51)	88 (22.34)	15 (3.81)	10 (2.54)	23 (5.84)	18 (4.57)	12 (3.05)	24 (6.09)	23 (5.84)	2 (0.51)
Pop‡	50,913,333 (100)	9,887,778 (19.42)	3,438,000 (6.75)	2,464,889 (4.84)	2,870,889 (5.64)	1,500,556 (2.95)	1,533,222 (3.01)	1,146,111 (2.25)	186,000 (0.37)	12,476,111 (24.50)	1,512,778 (2.97)	1,587,222 (3.12)	2,116,778 (4.16)	1,822,000 (3.58)	1,787,778 (3.51)	2,667,556 (5.24)	3,312,000 (6.51)	603,667 (1.19)

*Pop, population.
†Total number of patients during 2011–2019.
‡The average population per year and city, province, and country.

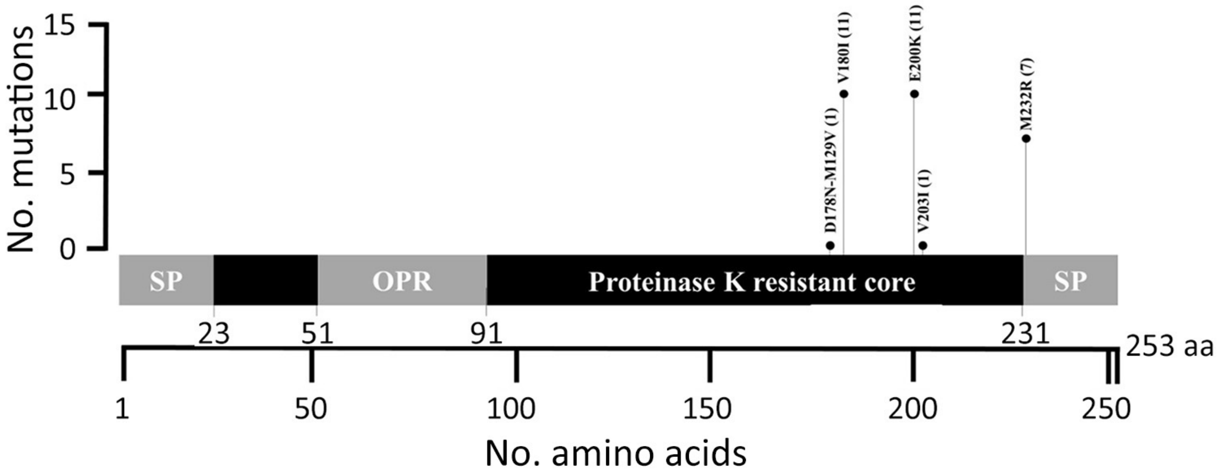

Appendix Figure 1. Schematic map of the human prion protein (PrP) with germline mutations of the prion protein gene (*PRNP*) found in familial Creutzfeld-Jakob disease patients, South Korea, 2001–2019. Parentheses indicate the number of patients carrying each germline mutation. OPR, octapeptide repeat region; SP, signal peptide.

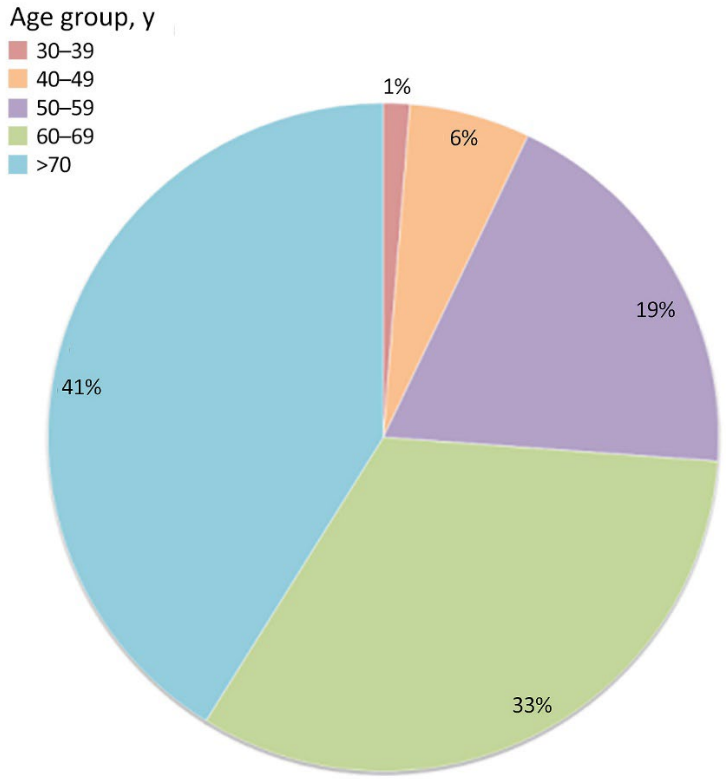

Appendix Figure 2. Distribution of Creutzfeldt-Jakob disease cases by age group, South Korea, 2001–2019.
